# COVID-19 and the Consequences of Anchoring Bias

**DOI:** 10.3201/eid2708.211107

**Published:** 2021-08

**Authors:** Harold W. Horowitz, Caren Behar, Jeffrey Greene

**Affiliations:** Weill Cornell Medicine, New York, New York, USA (H.W. Horowitz);; New York-Presbyterian Brooklyn Methodist Hospital, Brooklyn, New York, USA (H.W. Horowitz);; New York University Langone School of Medicine, New York (C. Behar, J. Greene)

**Keywords:** COVID-19, coronavirus disease, SARS-CoV-2, severe acute respiratory syndrome coronavirus 2, viruses, respiratory infections, zoonoses, Lyme disease, human granulocytic anaplasmosis, anchoring bias, vector-borne infections

## Abstract

Suspicion of coronavirus disease in febrile patients might lead to anchoring bias, causing misdiagnosis of other infections for which epidemiologic risks are present. This bias has potentially severe consequences, illustrated by cases of human granulocytic anaplasmosis and Lyme disease in a pregnant woman and human granulocytic anaplasmosis in another person.

Coronavirus disease (COVID-19) took the United States by force during the first quarter of 2020, affecting the economy, societal norms, and the delivery of medical care (*1*,*2*). As fear of COVID-19 has spread, diagnosing COVID-19 in febrile persons has been prioritized, and patients may be presumed to have COVID-19 pending results of testing for severe acute respiratory syndrome coronavirus 2 (SARS-CoV-2). This mindset has had unintended consequences, including delaying of evaluations for other infectious diseases, potentially leading to adverse outcomes. We describe 2 cases that illustrate this point.

In the first case, a 35-year-old man left New York, New York, USA, to go hiking in Maryland during June 5–June 7, 2020. He experienced fever, body aches, and fatigue during June 10–13 that resolved but left him fatigued and weak. He was seen on June 19; laboratory results were unremarkable, but lymphopenia was detected. He tested negative for SARS-CoV-2 on June 19 and June 25 by PCR. On June 25, ELISA for Lyme disease was positive, and reflex to Western blot revealed IgM 41-kD, 39-kD, and 23-kD bands but no IgG bands. Fever up to 38°C recurred on June 22 and lasted until June 29; he also experienced persistent fatigue and myalgia. Further testing on July 6 revealed serologic results for Lyme similar to results from June 25 and *Anaplasma phagocytophilum* titers of IgM 1:320 and IgG 1:1260. Anaplasma PCR was negative on that date. He was treated with doxycycline for 10 days and recovered.

In the second case, a 31-year-old woman who was 6 months pregnant left New York at the end of May 2020 to rent a house in Ulster County, New York. On June 3, she removed a tick from her neck. On June 9, she experienced severe headaches and the next day had low-grade fever, chills, and body aches. She had no cough, shortness of breath, or sore throat. On June 10, she tested negative for SARS-CoV-2 by PCR. She continued to have extreme fatigue, myalgia, and low-grade fever. She was prescribed oseltamivir by her obstetrician on June 11. On June 14, she felt better. Repeat PCR testing for SARS-CoV-2 on June 15 was negative. She continued to improve until June 23, when she experienced recurrent fever up to 38.9°C, chills, and lethargy. She contacted her obstetrician and was told she had a presumptive diagnosis of COVID-19. On June 30, she saw her internist and underwent laboratory testing for tickborne illnesses; she was treated empirically with amoxicillin because of her risks for Lyme disease. PCR for *A. phagocytophilum* was positive, as was a second test on July 8. Serologic results for Lyme were positive for 41-kD, 39-kD, and 23-kD bands with no IgG bands. Platelets were 140,000 (previously 336,000), aspartate aminotransferase was 95, and alanine aminotransferase was 81. Several weeks later, studies revealed anaplasma IgM 1:256 and IgG 1:1,280. Lyme disease C6 antibody was positive. After discussion, the patient and her physicians chose not to treat for anaplasmosis because she was clinically improving. The patient has remained well, and the child was born healthy by normal spontaneous vaginal delivery.

COVID-19 has had devastating effects on the medical system and led to widespread changes in the practice of medicine. We believe that the imperative to rule out COVID-19 led to diagnostic anchoring bias in these cases. Such biases are among the most common in the heuristic decision-making process (*3**,**4*). Of note, in these 2 cases (case 1, human granulocytic anaplasmosis [HGA]; case 2, co-infection with Lyme disease and HGA), COVID-19 was ruled out without considering other diagnoses, even though the patients were visiting areas to which tickborne diseases are endemic. Given the incidence of such diseases in these areas and widespread attempts to educate healthcare providers about these diseases, failure to evaluate for tickborne infections would be difficult to imagine before COVID-19. Although both of these patients have done well, serious consequences to the fetus could have occurred if Lyme disease had gone undiagnosed and untreated (*5*). Although transmission of *A. phagocytophilum* during pregnancy has been reported (*6*) and treatment during pregnancy in a limited number of cases has possibly prevented transmission (*7*), in this instance the patient cleared the anaplasma without treatment, and the child was born disease-free. Clearance of infection without treatment has been reported in other studies, but we are unaware of cases describing the outcome of pregnancy in untreated women with acute HGA (*8*).

We appreciate the devastating effects that a missed COVID-19 diagnosis can have on a person, as well as the epidemiologic implications thereof. However, failing to diagnose tickborne illnesses and other infections also can have serious consequences. Healthcare providers must keep an open mind to diagnoses other than COVID-19 in febrile patients and not fall prey to misdiagnosis because of current pressures to evaluate for COVID-19.

## References

[1] CDC. COVID-19 Response Team. Severe outcomes among patients with coronavirus disease 2019 (COVID-19)—United States, February 12–March 16, 2020. MMWR Morb Wkly Rep. 2020;69:343–6. 10.15585/mmwr.mm6912e2 PMC7725513 32214079

[2] Hollander JE, Carr BG. Virtually Perfect? Telemedicine for Covid-19. N Engl J Med. 2020;382:1679–81. 10.1056/NEJMp2003539 32160451

[3] Sapersnik G, Redelmeier D, Ruff CC, et a. Cognitive biases associated with medical decisions: a systematic review. BMC Med Inform Decis Mak. 2016;16:138. 10.1186/s12911-016-0377-1 27809908 PMC5093937

[4] Ogdie AR, Reilly JB, Pang WG, Keddem S, Barg FK, Von Feldt JM, et al. Seen through their eyes: residents’ reflections on the cognitive and contextual components of diagnostic errors in medicine. Acad Med. 2012;87:1361–7. 10.1097/ACM.0b013e31826742c9 22914511 PMC3703642

[5] Waddell LA, Greig J, Lindsay LR, Hinckley AF, Ogden NH. A systematic review on the impact of gestational Lyme disease in humans on the fetus and newborn. PLoS One. 2018;13:e0207067. 10.1371/journal.pone.0207067 30419059 PMC6231644

[6] Horowitz HW, Kilchevski E, Haber S, et al. Brief report: Perinatal transmission of the human granulocytic ehrlichiosis agent. N Engl J Med. 1998;339:375–8. 10.1056/NEJM199808063390604 9691104

[7] Dhand A, Nadelman RB, Aguero-Rosenfeld ME, Haddad F, Stokes D, Horowitz HW. Human granulocytic anaplasmosis in pregnancy: case series and review of literature. Clin Infect Dis. 2007;45:589–93. 10.1086/520659 17682993

[8] Bakken JS, Haller I, Riddell D, Walls JJ, Dumler JS. The serological response of patients infected with the agent of human granulocytic ehrlichiosis. Clin Infect Dis. 2002;34:22–7. 10.1086/323811 11731941

